# Colonoscopy Quality Indicators in Transition: From Adenoma Detection Rate to Serrated Lesion Detection and Beyond

**DOI:** 10.3390/diagnostics16020258

**Published:** 2026-01-14

**Authors:** Aryan Jain, James Javier, Kyle Nguyen-Ngo, Micheal Tadros

**Affiliations:** Department of Gastroenterology, Albany Medical College, Albany, NY 12208, USA; javierj@amc.edu (J.J.J.); nguyenky@amc.edu (K.N.-N.); tadrosm1@amc.edu (M.T.)

**Keywords:** colonoscopy quality, adenoma detection rate, sessile serrated lesions, adenomas per colonoscopy, colorectal cancer screening, image-enhanced endoscopy, computer-aided detection, colonoscopy quality metrics, sessile polyp detection rate

## Abstract

Colonoscopy is central to colorectal cancer (CRC) prevention, and its effectiveness is determined by the quality of mucosal inspection and lesion detection. The adenoma detection rate (ADR) remains the most widely validated quality benchmark due to its strong inverse association with interval CRC. However, reliance on ADR alone is increasingly recognized as insufficient, particularly given the growing understanding of the serrated neoplasia pathway, which contributes up to one-third of sporadic CRCs. This has driven the emergence of complementary metrics, such as the sessile polyp detection rate (SPDR) and adenomas per colonoscopy (APC). Although SPDR offers important advantages for capturing serrated pathology, challenges persist, including interobserver variability, inconsistent pathology thresholds, limited endoscopist training, and the absence of standardized benchmarks. Alongside these evolving metrics, technological advancements such as image-enhanced endoscopy, computer-aided detection, high-definition optics, and distal attachment devices have demonstrated measurable improvements in detecting subtle lesions and reducing operator-dependent variability. Large real-world registries, including GIQuIC, now support the development and validation of composite models that integrate ADR, SPDR, and APC to better reflect the full spectrum of neoplasia detection. As the field advances, redefining colonoscopy quality will require reconciling established metrics with newer indicators that more comprehensively address both conventional adenomas and serrated lesions.

## 1. Introduction

Colorectal cancer (CRC) is one of the leading causes of cancer-related morbidity and mortality worldwide. Colonoscopy remains the cornerstone of CRC prevention and early detection. It not only identifies established cancers but also plays a therapeutic role by enabling the detection and removal of precancerous lesions during the same procedure [1]. Most CRCs develop from benign precursor lesions known as colorectal polyps. Colorectal polyps are abnormal mucosal growths that protrude into the lumen of the colon [2]. While many are benign, certain types have malignant potential and serve as precursors to CRC. The process by which benign polyps evolve into invasive cancer is known as the adenoma-carcinoma sequence or, in some cases, the serrated neoplasia pathway [3].

There are two major classes of polyps implicated in CRC pathogenesis: conventional adenomas and serrated lesions. Conventional adenomas, which include tubular, tubulovillous, and villous histologic subtypes, are the most common and follow the traditional progression of accumulating genetic mutations that lead to dysplasia and eventual carcinoma [4]. These polyps are generally more prominent endoscopically and are commonly found in the distal colon and rectum. Serrated polyps, which include hyperplastic polyps, sessile serrated lesions (SSLs), and traditional serrated adenomas (TSAs), represent an alternative route to cancer, particularly for right-sided and interval CRCs. SSLs, in particular, can be flat, covered in mucous, and easily missed during a colonoscopy [4]. They follow a molecular pathway characterized by BRAF mutations and CpG island methylator phenotype (CIMP), leading to silencing of tumor suppressor genes [5]. Failure to identify these polyps contributes to the development of interval cancers—cancers that arise after a negative colonoscopy but before the next recommended screening [6]. As such, identifying both adenomas and serrated lesions during colonoscopy is not only diagnostic but fundamentally preventative.

To ensure effectiveness, colonoscopy performance is now routinely measured using specific quality indicators. Among these, adenoma detection rate (ADR) has emerged as a validated benchmark strongly correlated with interval cancer risk. ADR is defined as the proportion of screening colonoscopies in which at least one adenoma is found [7]. Higher ADR corresponds with a lower likelihood of post-colonoscopy cancer, making it an essential benchmark for endoscopist performance. Similarly, the serrated polyp detection rate (SPDR) has also emerged as an additional metric. Serrated lesions are often more challenging to detect due to their flat morphology, subtle borders, and frequent location in the proximal colon [8]. As a result, they are more likely to be missed during a routine colonoscopy. Incorporating SPDR along with ADR provides a more comprehensive measure of exam quality.

Together, these detection-based metrics serve not only as tools for quality assurance but also as critical drivers of innovation in endoscopic technique, training, and technology. Despite the widespread adoption of colonoscopy, detection variability between providers remains substantial. In response, numerous technological innovations, such as artificial intelligence (AI)-assisted detection and advanced imaging techniques, have been developed to optimize quality. This review summarizes current standards in colonoscopy quality, with a particular focus on adenoma and serrated lesion detection. We highlight recent advancements in detection techniques, review the evidence behind benchmark metrics, and discuss strategies to improve performance and reduce the incidence of colorectal cancer. This narrative review was informed by a targeted literature search of PubMed and MEDLINE databases, focusing on English-language studies published from 2000 through 2025. Key search terms included colonoscopy quality, adenoma detection rate, serrated polyp detection, and colonoscopy performance metrics. Relevant guidelines, landmark studies, and recent systematic reviews were prioritized.

## 2. Adenoma Detection Rate

**Figure 1:**
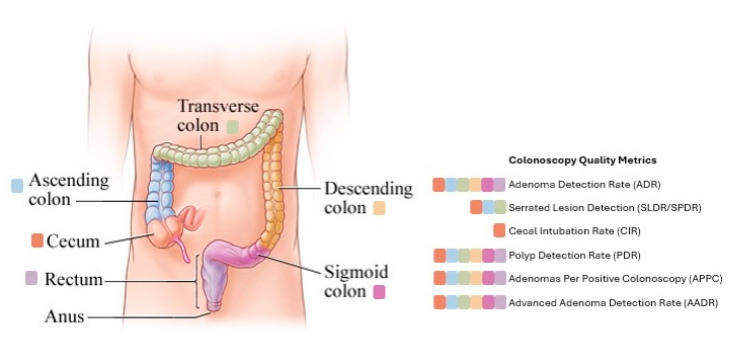
Anatomic regions of the colon corresponding to key colonoscopy quality indicators.

With each colonoscopy, there are several variations and factors that contribute to a quality procedure. As a result, quality indicators were created to objectively measure an endoscopist’s performance. A summary of major colonoscopy quality indicators, including definitions, benchmarks, strengths, and limitations, is provided in Table 1. These quality indicators vary by anatomic region of the colon, as illustrated in Figure 1. ADR is widely recognized as the most important and validated quality indicator in colonoscopy. ADR reflects both the thoroughness of mucosal inspection and the endoscopist’s ability to recognize subtle neoplastic lesions. Numerous studies have shown that ADR is inversely associated with the risk of interval colorectal cancer [9]. There are two specific landmark studies that firmly established ADR as a predictor of patient outcomes. In a large screening cohort, one study found that patients whose colonoscopies were performed by endoscopists with ADRs <20% faced a 10- to 12-fold higher risk of interval CRC compared to those with ADRs ≥20% [10]. Similarly, another large retrospective study demonstrated that each 1% increase in ADR was associated with a 3% reduction in the risk of interval CRC, advanced-stage CRC, and cancer-related mortality [7]. These findings led prominent societies such as the U.S. Multi-Society Task Force on Colorectal Cancer to establish minimum ADR benchmarks of 25% overall, with sex-specific thresholds of 30% for men and 20% for women [11].

Beyond its prognostic significance, ADR also reflects important epidemiologic trends. As adenoma prevalence has increased, the American Society for Gastrointestinal Endoscopy (ASGE) now recommends measuring ADR in patients aged ≥45 years and suggests a minimum performance target of 35%, with sex-specific thresholds of 40% for men and 30% for women [12]. This is based on accumulating data showing that ADRs have risen steadily over the past decade, with a mean of 38% in U.S. practices by 2018, and that the risk of PCCRC (post-colonoscopy colorectal cancer) continues to decrease with ADRs above 35–40% [13]. A recent large-scale retrospective study of nearly 19,000 average-risk individuals in rural China revealed that ADR increases significantly after the age of 45, especially in men, lending support to the revised CRC screening guidelines that recommend initiating screening at age 45 [14]. However, while ADR is a reliable measure of detection, it does not capture all dimensions of endoscopic performance.

One major limitation of ADR is its binary structure: it registers whether at least one adenoma is found but provides no information about the total number of adenomas, their location, or whether they were completely resected. As a result, it is vulnerable to the “one and done” phenomenon, in which an endoscopist may reduce inspection intensity after identifying a single lesion [15]. It also fails to measure adenoma removal quality, which is equally critical for CRC prevention. Furthermore, ADR can be influenced by external factors such as patient demographics, referral population, and even interobserver variability in histopathologic diagnosis [9].

To address these shortcomings, complementary metrics have been proposed. One such measure, Adenoma Under the Curve (AUC), incorporates both the rate of adenoma detection and the number of adenomas per colonoscopy. In one comparative study, AUC revealed a 25% performance gap between academic and community settings, despite only a 10% difference in ADR, suggesting it may better capture the depth of mucosal inspection [9]. Additional metrics such as advanced ADR (focused on high-risk adenomas) and adenoma per colonoscopy (APC) are under investigation, though none have yet surpassed ADR as the core benchmark.

Technological advances such as Endocuff-assisted colonoscopy and linked color imaging (LCI) have been shown to significantly improve ADR across a range of practice settings [16]. Among these, AI-assisted systems appear particularly promising for standardizing detection performance and reducing inter-operator variability. As colonoscopy continues to evolve with the integration of real-time computer-aided detection and enhanced imaging tools, ADR remains a foundational metric.

## 3. Serrated Lesion Detection (SPDR)

While conventional adenomas have long been the primary focus of colonoscopy quality improvement efforts, there is growing recognition that serrated lesions, particularly sessile serrated lesions and traditional serrated adenomas (TSAs), contribute significantly to colorectal cancer burden, especially in the proximal colon [17]. Serrated polyps are now understood to be responsible for up to 20–30% of sporadic CRCs [18]. Unlike adenomas, serrated lesions often exhibit a flat morphology, indistinct borders, and a mucus cap, making them easy to miss during routine inspection.

To address this diagnostic blind spot, the serrated polyp detection rate (SPDR) has emerged as an important, though still evolving, quality metric. SPDR is typically defined as the proportion of colonoscopies in which at least one serrated lesion (usually an SSL or TSA) is identified. Although benchmark thresholds have not been as well established compared to those for ADR, recent literature suggests target SPDR values ranging from 5 to 10% may reflect adequate detection skill [19]. The establishment of universal SPDR benchmarks is inherently challenging due to substantial heterogeneity in serrated lesion prevalence across patient populations, procedural indications, and colonic segments, which limits the generalizability of fixed threshold targets. Variability in SPDR has been observed across endoscopists, sometimes exceeding the variability seen with ADR, raising concern that serrated lesions are being inconsistently detected and under-reported [20]. This inconsistency is particularly problematic given that SSLs account for 20–30% of sporadic colorectal cancers and disproportionately contribute to interval cancers due to their flat morphology and subtle endoscopic features.

Despite its promise, several limitations hinder widespread SPDR adoption. A key challenge in implementing SPDR as a standard quality metric lies in the interobserver variability of serrated lesion diagnosis by pathologists, as well as limited awareness and training among endoscopists to recognize subtle endoscopic features. Moreover, studies have shown that SPDR is not strongly correlated with ADR, reinforcing the need for a separate, dedicated focus on serrated lesion detection during colonoscopy [21]. Population-level factors such as referral bias, case mix, and demographic differences may further influence SPDR, contributing to wide performance dispersion and complicating direct comparisons between endoscopists and practice settings [22]. Still, serrated lesion detection is clinically critical, as patients with SSLs, particularly proximal or ≥10 mm lesions, face a higher risk of future advanced neoplasia [23]. Improvements in SPDR, therefore, have the potential to significantly enhance colonoscopy’s preventive effectiveness. Emerging strategies, including prolonged right colon withdrawal, retroflexion, enhanced imaging modalities such as NBI, TXI, or Linked Color Imaging, and AI-assisted detection systems, have shown promise in improving serrated lesion recognition, although their implementation remains inconsistent and requires further guideline standardization [24,25,26]. To overcome benchmark variability, large registry-based datasets may enable risk-adjusted or percentile-based SPDR modeling that accounts for patient demographics, indication, bowel preparation quality, and procedural factors. Looking ahead, future directions include the development of composite quality models integrating ADR, SPDR, and adenomas per colonoscopy into weighted indices that better reflect both major colorectal carcinogenesis pathways [27]. Large-scale registries such as GIQuIC will be crucial for validating SPDR benchmarks, supporting longitudinal evaluation of interventions, and standardizing data collection to reduce variability in lesion classification [28]. These efforts will be essential before SPDR can be routinely adopted as a national quality indicator alongside ADR.

## 4. Cecal Intubation Rate (CIR)

Complete visualization of the colon is fundamental to the effectiveness of colonoscopy as a screening and diagnostic tool. The cecal intubation rate (CIR) measures the proportion of colonoscopies in which the endoscopist successfully advances the colonoscope to the cecum, typically confirmed by identifying the appendiceal orifice and ileocecal valve, as shown in Figure 1. The CIR is one of the consistently validated quality indicators because incomplete examinations correlate with missed proximal neoplasia, higher rates of interval colorectal cancer, and the need for early repeat procedures [28]. High CIR reflects adequate technique in navigating tortuous or angulated anatomy and the ability to maintain a luminal view through suctioning, insufflation, and position changes. Studies have shown that incomplete colonoscopies are significantly associated with an increased risk of proximal interval CRC, which highlights the importance of achieving complete examination when possible [29].

After the recognition of CIR as an important colonoscopy quality indicator, clear benchmarks were established. For screening and surveillance colonoscopies, the recommended minimum CIR is ≥95% [27]. Lower thresholds may be allowed for purely diagnostic procedures where patient factors such as obstructing tumors or severe colitis limit advancement. However, these cases should be carefully documented to distinguish technical limitations from operator performance. Persistent CIR values below benchmark levels require review of case logs, technical training, or evaluation of outside contributors, such as bowel preparation quality.

Despite its strengths, the CIR is not a comprehensive measure of colonoscopy quality. A technically complete examination does not guarantee adequate mucosal inspection, particularly if the endoscopist advances rapidly, fails to control looping, or does not optimize visualization during insertion. The CIR can also be influenced by factors outside of operator control, such as bowel preparation, patient discomfort thresholds, and institutional support for adequate sedation. Nonetheless, as colonoscopy technology and adjunctive tools have improved, CIR has become a valuable metric, with contemporary practices consistently achieving rates above 95% [28].

## 5. Polyp Detection Rate

Polyp detection rate (PDR) is defined as the proportion of colonoscopies in which at least one polyp of any histologic type is identified [30]. Although less specific than adenoma detection rate (ADR), PDR remains a widely used metric in complement to ADR because it does not require pathology correlation and is a quick estimate of mucosal inspection quality. PDR also covers a broader range of detected lesions, including hyperplastic polyps, inflammatory polyps, and serrated lesions, all of which may be clinically relevant depending on location and size.

The primary value of PDR is its practicality. Metrics that require pathology, such as ADR, require accurate integration of histologic data, which is not reliably feasible across all practice settings. PDR relies solely on endoscopic documentation and enables immediate monitoring and comparison of detection performance [30]. Multiple studies show a strong correlation between PDR and ADR, making PDR a reasonable substitute for ADR in settings where pathology data are incomplete [31]. This correlation allows institutions to flag potential underperformance earlier while awaiting confirmed histologic metrics.

Despite its utility, PDR has significant limitations. Because it includes all polyp types, it is vulnerable to inflation through the removal or documentation of clinically insignificant lesions. This weakness makes it less reliable as a true cancer-prevention indicator. Additionally, PDR does not distinguish between adenomatous and serrated lesions and does not account for the total number of lesions found during an examination [32]. As a result, PDR does not meaningfully reflect the thoroughness of mucosal inspection or lesion recognition complexity. The metric also tends to be less stable across varying patient populations because polyp prevalence is influenced more strongly by demographic factors than adenoma prevalence.

Benchmark values for PDR vary across studies and institutions, but many programs consider 40% an expected minimum in average-risk screening populations, with higher targets in practices that have fully adopted high-definition imaging [31]. Nonetheless, PDR should not be interpreted in isolation. It functions best as an adjunctive measure that supports broader quality monitoring, identifies trends in detection behavior, and provides rapid-feedback signals when histology-linked metrics are unavailable. When interpreted alongside other metrics, PDR helps create a more comprehensive picture of endoscopic detection performance while avoiding excessive reliance on a single indicator.

## 6. Adenomas per Positive Colonoscopy

Adenomas per positive colonoscopy (APPC) represents the average number of adenomas identified among colonoscopies in which at least one adenoma is found. Unlike adenoma detection rate (ADR), which only captures whether the endoscopist identified any adenoma, APPC provides a quantitative assessment of mucosal inspection depth while avoiding the extreme variability seen with metrics that average adenoma counts across all procedures, including negative exams [33]. By focusing solely on examinations where at least one adenoma was detected, APPC reduces statistical interference and offers a more stable estimate of true detection performance.

The strength of APPC is its ability to describe differences in lesion-finding behavior that ADR alone conceals. Two endoscopists may have identical ADRs yet differ substantially in how thoroughly they evaluate the colon once the first adenoma is found. Studies have shown that APPC correlates more strongly than ADR with metrics tied to overall detection burden, such as adenomas per colonoscopy [34]. APPC may more effectively reveal underperformance in experienced endoscopists for whom ADR meets minimum thresholds but whose inspection technique remains suboptimal [34].

Nonetheless, APPC is not without its limitations. Because it excludes negative colonoscopies entirely, it artificially narrows the dataset and may overrepresent endoscopists who work with higher-risk populations. This selective averaging can obscure meaningful differences in patient mix and does not reflect the full procedural portfolio of the operator. APPC also shares the vulnerabilities of other count-based metrics in that it can be influenced by over-removal of clinically insignificant diminutive lesions and is sensitive to pathology interpretation consistency. Additionally, benchmarks for APPC remain undefined, with no consensus existing on threshold targets, limiting its use as a stand-alone metric.

## 7. Advanced Adenoma Detection Rate

The advanced adenoma detection rate (AADR) measures the proportion of colonoscopies in which at least one advanced adenoma is detected. Advanced adenomas are typically defined as adenomas ≥10 mm, those with villous or tubulovillous histology, or those demonstrating high-grade dysplasia [35]. Because these lesions represent the subset of precancerous polyps with the highest malignant potential, AADR is an assessment of an endoscopist’s ability to detect pathology that most directly influences colorectal cancer prevention.

The AADR’s strength is its clinical significance. Multiple studies have shown that the presence of advanced adenomas strongly predicts future advanced neoplasia and colorectal cancer, making their detection a critical objective of screening colonoscopy [36]. By isolating clinically consequential lesions, AADR complements broader detection metrics like ADR. AADR provides a more outcome-driven approach, emphasizing detection of the lesions most important to long-term patient risk stratification [37].

Despite its conceptual value, AADR is limited by substantial statistical and practical constraints. Advanced adenomas are relatively uncommon, which leads to significant variability in measured rates, especially for low-volume endoscopists. As a result, AADR is more sensitive to random variation than ADR, and year-over-year comparisons often lack stability [38]. Additionally, AADR is heavily influenced by patient demographics, referral patterns, and underlying population risk. Endoscopists serving older or higher-risk patient populations may appear to have superior AADR, independent of technique, whereas others practicing in younger or lower-risk settings may struggle to meet expected ranges despite adequate performance.

## 8. Withdrawal Time

Withdrawal time is one of the most validated surrogate markers of colonoscopy quality, with numerous studies demonstrating a strong, positive correlation between longer withdrawal times and higher adenoma detection rates. For example, a landmark analysis found that each additional minute of withdrawal time was associated with a 3.6% increase in ADR [39]. Reflecting on this evidence, leading professional societies recommend a minimum withdrawal time of 6 min (excluding time spent on interventions like polyp removal) to ensure comprehensive mucosal inspection [40]. Adherence to this 6 min standard is associated with a reduction in the incidence of interval colorectal cancer, underscoring its importance for preventive effectiveness [41]. Notably, studies have shown that extending withdrawal time beyond 6 min, particularly up to 9 min, can yield even greater improvements in ADR, especially in the proximal colon and among less experienced endoscopists [42]. Although a 6 min withdrawal serves as a widely acknowledged baseline, it may need to be exceeded in higher-risk scenarios, such as patients with a history of adenomas or suboptimal bowel preparation, to maximize detection and preventive impact.

## 9. Current Barriers to Quality Colonoscopies

Despite standardized guidelines for colonoscopy performance, inter-endoscopist variability remains a major barrier to consistent quality. To that end, prior studies have demonstrated that ADR can significantly differ between practitioners even within the same institution. This variation reflects differences in not only expertise, such as withdrawal technique and thoroughness with mucosal inspection, but also personal preference, like utilization of adjunctive technologies. The basis of such variability is multifactorial; differences may stem from training background and procedural volume, with higher-volume endoscopists often achieving higher ADR yield [7,43]. Institutional culture can also play a role, as centers with robust quality monitoring programs often display less inter-practitioner variability compared to those lacking structured feedback systems. Furthermore, even when lesions are resected, discrepancies in pathology interpretation can undermine quality. For instance, a histologic distinction between hyperplastic polyps and SSLs is often subtle, and inter-pathologist agreement is imperfect [22].

Other barriers to colonoscopy quality arise from factors that extend beyond the endoscopist’s direct technical skill. Time pressure and scheduling demands can influence behavior in subtle but important ways. High procedural volumes and productivity pressures have been associated with shortened withdrawal times and less meticulous mucosal inspection, which are both linked to lower adenoma detection rates [44,45]. Inadequate bowel preparation remains one of the most significant obstacles to effective screening. Poor preparation reduces mucosal visibility, particularly in the right colon, where flat lesions and retained debris are more common. This is consistently associated with higher miss rates for advanced neoplasia [46]. Preparation quality is highly variable and may be impacted by patient-related factors such as adherence, comorbidities (e.g., constipation, diabetes, or opioid use), and the type of regimen prescribed [47]. Suboptimal preps often necessitate repeat colonoscopies, which drive up costs, increase patient inconvenience, and undermine screening effectiveness. In conjunction with poor bowel preparation, patient-specific factors and anatomical variability inherently introduce additional challenges. Prior abdominal surgeries, particularly gastrectomies and hysterectomies, were associated with a lower cecal intubation rate and incomplete procedures [48]. Additionally, several studies have also suggested a potential U-shaped relationship between central adiposity, as represented by body mass index (BMI), and relative procedural difficulty. Specifically, both high and low BMIs are positively correlated with colonoscopy times and incompleteness [49].

Fatigue and human factors also play a meaningful role. Endoscopist fatigue, whether from extended procedure lists, overnight call duties, or cognitive overload, has been associated with impaired performance, including lower ADRs, poorer withdrawal quality, and even higher complication rates [50]. These findings emphasize that quality is not solely a matter of training or vigilance, but also of managing systemic pressures and human limitations. Addressing these barriers requires both institutional support and ongoing monitoring of quality metrics.

## 10. Strategies to Improve Quality Parameters

Standardized training programs, integrating both didactic instruction and hands-on practical experience, play a vital role in reducing inter-endoscopist variability and reinforcing consistent colonoscopy techniques. For example, an endoscopic quality improvement initiative demonstrated that structured training combined with personalized feedback led to an absolute increase of approximately 11% in ADR across practitioners [51]. Such programs are particularly important for improving the detection of subtle lesions like sessile serrated lesions and flat adenomas, which are frequently associated with interval cancers. By emphasizing lesion recognition and lesion resection quality, these interventions help close gaps in preventive performance. Furthermore, routine auditing and continuous feedback against quality metrics, such as ADR and withdrawal time, enable endoscopists to compare their performance against institutional standards and national benchmarks [52].

While detection-based metrics such as ADR and SPDR are central to colonoscopy quality assessment, they represent only one component of effective colorectal cancer prevention. Complete and durable resection of identified lesions is equally critical, as incomplete polyp removal has been implicated as a major contributor to interval colorectal cancer [10,51]. Prior studies have demonstrated that residual or recurrent neoplasia at polypectomy sites, particularly following removal of large or sessile lesions, accounts for a substantial proportion of post-colonoscopy cancers. As a result, quality indicators related to resection technique, margin assessment, and surveillance adherence are increasingly recognized as complementary measures of procedural effectiveness [3,6,35].

In addition, post-polypectomy outcomes, including adverse events, recurrence rates, and long-term associations with interval colorectal cancer, provide important downstream validation of colonoscopy quality [35]. Although these outcome-based metrics are more challenging to standardize and require longitudinal follow-up, they represent clinically meaningful endpoints that link procedural performance to patient-centered outcomes. Integrating detection metrics with measures of resection quality and long-term outcomes may therefore offer a more comprehensive framework for assessing colonoscopy quality.

The right colon is a frequent site for missed lesions, largely due to its complex anatomy and higher prevalence of flat serrated lesions. Retroflexion following cecal intubation provides an alternate viewing angle, uncovering lesions hidden behind folds or flexures. In a multicenter randomized trial, a second examination of the right colon, whether by retroflexion or a second forward view, improved ADR by approximately 11%, without significant procedural complications and with minimal additional time (about 1.6 min) [53]. Similarly, a 2022 study reported that this maneuver increased right-sided ADR by 5.5% [54]. Despite the potential benefits, the practice of retroflexion is not universally adopted, likely due to concerns over mucosal trauma and increased procedure time. However, data show a high success rate in performing retroflexion (up to 91.9%) and an exceptionally low adverse event rate of just 0.03% [55]. Thus, with proper training, retroflexion is both safe and time-efficient, and can be a valuable adjunct, especially in high-risk patients. Other challenging segments, such as the hepatic flexure and sigmoid colon, may also benefit from second-look strategies, which can be conducted immediately following cecal intubation or the withdrawal phase. Though adopting second-look strategies slightly increases procedural times, such strategies have consistently shown measurable improvements in ADR and hold promise for reducing interval colorectal cancers [56].

## 11. Enhanced Imaging and Technical Modifications

Several technical innovations have been developed to overcome the limitations of standard colonoscopy and to improve lesion detection. Distal attachment devices have been developed to improve mucosal visualization by mechanically altering the field of view during colonoscopy. The most established of these is transparent cap-assisted colonoscopy (CAC), in which a clear cap is affixed to the distal end of the colonoscope. The cap maintains a uniform distance between the scope and mucosa, flattens folds, and prevents mucosal collapse into the lens. Randomized trials and meta-analyses demonstrate that CAC not only facilitates cecal intubation by preventing mucosal collapse into the lens but also modestly increases ADR and reduces intubation times [57,58].

Building on this principle, Endocuff-assisted colonoscopy (EAC) employs a disposable cap with flexible projections that fold back during insertion and splay outward on withdrawal. Endoscopic attachments, such as the Endocuff Vision and transparent caps, are simple mechanical devices designed to optimize mucosal visualization. Both function by flattening folds and stabilizing the distal tip of the scope, thereby exposing mucosa that might otherwise remain hidden. Randomized controlled trials have demonstrated that Endocuff-assisted colonoscopy improves ADR, particularly in the proximal colon, where folds and flexures are more prevalent [59,60]. These devices are especially valuable in patients with challenging anatomy, including those with a tortuous or redundant colon, representing a refinement of earlier transparent cap technology. Collectively, distal attachment devices highlight how simple mechanical innovations can enhance inspection thoroughness and reduce the risk of missed lesions.

Water-aided techniques represent another approach, where water is used instead of air to distend the lumen and improve mucosal visibility. This is done by washing debris and minimizing bubbles. In particular, the water-exchange method permits cleansing and inspection simultaneously during insertion. It has been shown to improve ADR, reduce patient discomfort, and lower sedation requirements, albeit with slightly longer insertion times [24,61]. Chromoendoscopy is another valuable asset for lesion recognition, especially in patients at increased risk. It encompasses both dye-spray and virtual modalities and is a widely studied adjunct aimed at improving detection beyond standard white-light endoscopy. Dye-based methods enhance mucosal contrast and have been shown to improve neoplasia detection compared with white-light endoscopy [62]. Beyond traditional dye-spray, virtual chromoendoscopy technologies, such as linked color imaging (LCI) and narrow band imaging (NBI), leverage altered light spectra and digital post-processing to highlight subtle lesions. Recent evidence indicates that these tools enhance the detection of SSLs and aid in delineating lesion margins prior to resection [63]. Together, these techniques underscore how adjunctive technologies can augment mucosal visualization, facilitate thorough inspection, and ultimately improve the preventive effectiveness of colonoscopy.

## 12. Role of Artificial Intelligence in Colonoscopy Quality Metrics

Advancements in endoscopic technology are continually introducing innovative modalities designed to minimize limitations in colonoscopy performance and maximize patient outcomes. Computer-aided detection (CADe) systems integrate artificial intelligence into colonoscopy workflows to assist with real-time polyp identification. Several studies have shown that CADe can significantly improve ADR across varying levels of endoscopist experience, helping buffer inter-operator variability [64,65]. While CADe offers consistency and reduces fatigue-related performance declines, its heightened sensitivity often results in false positives due to stool or bubbles, and necessitates investment in compatible imaging systems and workflow integration [66]. Alongside CADe is computer-aided diagnosis (CADx), an AI-powered model designed to systematically differentiate between adenomatous and non-adenomatous diminutive polyps in real time. CADx may be leveraged as an optical biopsy to guide resection decisions. Prior research has revealed that in approximately 90% of patient cases, CADx-informed polypectomies were confirmed to be in accordance with histology guidelines. CADx would further contribute to maximizing endoscopist objectivity, as well as reducing the burden of unnecessary polypectomies for patients [67].

Another application of AI lies in the assessment of bowel preparation, as suboptimal preparation significantly reduces ADR yield. After being trained with the Boston Bowel Preparation Scale (BBPS), AI systems have demonstrated success in evaluating preparation thoroughness and recommending re-cleansing as needed, ultimately manifesting in a reduced adenoma miss rate (AMR) [68]. Furthermore, real-time quality tracking systems such as the Automatic Quality Control System (AQCS) represent an emerging frontier. AQCS has been demonstrated in a randomized controlled trial to increase ADR, enhance withdrawal technique, and improve bowel preparation metrics through intra-procedural feedback [69,70]. Collectively, these innovations demonstrate how AI is destined to transform colonoscopy into a more standardized, data-driven, and optimized procedure.

Despite these demonstrated benefits, the widespread adoption of AI-assisted detection and advanced imaging technologies remains constrained by cost, infrastructure requirements, and workflow integration challenges, particularly in community-based practices. Although AI-assisted and technology-enhanced colonoscopy may improve detection, limitations related to false positives, workflow integration, training demands, and cost-effectiveness, particularly in community and lower-resource settings, should be considered when evaluating broader adoption. Upfront capital investment, software licensing, hardware compatibility, and the need for endoscopist and staff training may limit accessibility outside of high-resource or academic centers, raising concerns about uneven adoption across practice settings [66,71]. Integration into existing workflows may also introduce challenges related to alert fatigue, procedure efficiency, and interoperability with endoscopy reporting systems [64,66]. Addressing these barriers through scalable platforms, registry-supported implementation studies, and cost-effectiveness analyses will be essential to ensure that technological advances in colonoscopy quality do not inadvertently widen disparities in colorectal cancer prevention.

## 13. Generalizability Across Practice Settings

While much of the evidence supporting colonoscopy quality metrics and advanced detection technologies is derived from large academic centers, national registries, and high-resource healthcare systems, the translation of these approaches to community-based and lower-resource practice settings requires careful consideration. Community practices perform the majority of screening colonoscopies worldwide and may face constraints related to procedure time, staffing, access to advanced imaging platforms, and artificial intelligence tools. In these settings, foundational quality measures such as adequate bowel preparation, complete examination to the cecum, sufficient withdrawal time, and adherence to surveillance guidelines remain critical in preventive effectiveness. Lastly, scalable quality improvement strategies may offer practical pathways to improving colonoscopy quality across diverse healthcare environments.

## 14. Conclusions

Colonoscopy remains the cornerstone of colorectal cancer prevention, but its effectiveness is directly tied to the quality of mucosal inspection and lesion detection. The ADR has long served as the most validated quality benchmark, strongly linked to interval cancer risk and widely adopted in practice. However, ADR alone is insufficient to capture the full spectrum of colorectal neoplasia. The growing recognition of the serrated pathway, which accounts for up to a third of sporadic CRCs, has led to the emergence of the SPDR as a complementary metric. Despite its promise, SPDR faces barriers including interobserver variability, inconsistent benchmarks, and variable adoption across practices.

Looking forward, colonoscopy quality assessment will likely need to evolve from reliance on single metrics toward multidimensional frameworks that better reflect inspection thoroughness, lesion burden, and procedural completeness. Composite approaches incorporating ADR, SPDR, adenomas per colonoscopy, and withdrawal time may provide a more nuanced and clinically meaningful evaluation of endoscopist performance. Despite their utility, colonoscopy quality metrics remain the subject of ongoing debate. Concerns include the potential for metric-driven behavior, increasing administrative burden associated with monitoring multiple indicators, and uncertainty regarding whether incremental improvements in detection metrics consistently translate into meaningful patient-centered outcomes. Large-scale registries and standardized pathology reporting will be essential to validating these models and establishing generalizable benchmarks across diverse practice settings.

Conceptually, colonoscopy quality metrics may be best understood within a tiered framework. Foundational measures such as cecal intubation rate, adequate bowel preparation, and withdrawal time ensure procedural completeness. Detection-based metrics such as ADR and SPDR assess inspection quality across both adenomatous and serrated pathways. Finally, outcome-linked measures, including adenomas per colonoscopy and interval colorectal cancer rates, provide downstream validation of clinical effectiveness. Within this model, ADR remains a necessary baseline benchmark, while complementary metrics may be selectively emphasized based on patient population, practice setting, and available resources. Importantly, colonoscopy quality assessment should also account for patient-centered outcomes, including procedural burden, complication risk, anxiety related to surveillance intensity, and the role of shared decision-making. Aligning quality indicators with value-based care principles will be essential to ensuring that quality improvement efforts translate into meaningful benefits for patients.

Technological innovations, including image-enhanced endoscopy, distal attachment devices, and artificial intelligence-assisted detection, are poised to further reduce inter-operator variability and improve detection of subtle lesions. As these tools become increasingly integrated into routine practice, quality metrics must evolve in parallel to ensure that performance assessment remains outcome-driven and clinically relevant. Ultimately, refining colonoscopy quality evaluation through evidence-based, integrated frameworks has the potential to enhance consistency, equity, and preventive effectiveness in colorectal cancer screening.

## Figures and Tables

**Table 1:** Established and emerging colonoscopy quality metrics: definitions, benchmarks, strengths, and limitations.

Metric	Definition	Benchmark	Strengths	Limitations
Adenoma Detection Rate (ADR)	Percentage of screening colonoscopies with ≥1 adenoma	≥20–30%	ADR is a reliable predictor of patient outcomes and a significant epidemiological metric.	Does not note the total number or status of resection of adenomas
Serrated Lesion Detection (SPDR)	Percentage of colonoscopies with ≥1 serrated lesion	≥5–10%	Serrated lesions significantly contribute to colorectal cancer and SPDR is not strongly correlated with ADR	Variability in SPDR is high and training in recognizing serrated lesions is limited
Cecal Intubation Rate (CIR)	Percentage of colonoscopies where the scope is successfully advanced to the cecum	≥95%	Failure to reach the cecum can result in missed lesions and adenomas	It is not a detection metric like ADR or SPDR
Polyp Detection Rate (PDR)	Percentage of colonoscopies with ≥1 polyp	≥40%	Correlates well with ADR	Does not account for pathology so PDR may be over-reported
Adenomas Per Positive Colonoscopy (APPC)	Mean number of adenomas per positive colonoscopy	N/A	Reflects detection across the whole colon, not just the first polyp found	Incentivizes removal of clinically trivial lesions and does not have an accepted benchmark
Advanced Adenoma Detection Rate (AADR)	Proportion of colonoscopies where advanced adenomas (≥10 mm, villous histology, or high-grade dysplasia) are found	N/A	Focuses on finding clinically consequential neoplasia	Does not account for advanced serrated lesions and does not have an accepted benchmark
Withdrawal Time	Average time spent inspecting the mucosa during scope withdrawal (excluding time for polypectomy or therapeutic maneuvers)	≥6 min	Strongest surrogate marker for mucosal inspection quality; strongly correlates with higher ADR and lower interval CRC risk	Can be artificially extended without improving technique; may require >6 min in high-risk patients or with suboptimal prep; operator-level variation

## Data Availability

No new data were created or analyzed in this study. Data sharing is not applicable to this article.
